# The neural substrates of deliberative decision making: contrasting effects of hippocampus lesions on performance and vicarious trial-and-error behavior in a spatial memory task and a visual discrimination task

**DOI:** 10.3389/fnbeh.2012.00070

**Published:** 2012-10-30

**Authors:** David Bett, Elizabeth Allison, Lauren H. Murdoch, Karola Kaefer, Emma R. Wood, Paul A. Dudchenko

**Affiliations:** ^1^Psychology, School of Natural Sciences, University of StirlingStirling, UK; ^2^Centre for Cognitive and Neural Systems, School of Biomedical Sciences, University of EdinburghEdinburgh, UK

**Keywords:** hippocampus, vicarious trial-and-error, VTE

## Abstract

Vicarious trial-and-errors (VTEs) are back-and-forth movements of the head exhibited by rodents and other animals when faced with a decision. These behaviors have recently been associated with prospective sweeps of hippocampal place cell firing, and thus may reflect a rodent model of deliberative decision-making. The aim of the current study was to test whether the hippocampus is essential for VTEs in a spatial memory task and in a simple visual discrimination (VD) task. We found that lesions of the hippocampus with ibotenic acid produced a significant impairment in the accuracy of choices in a serial spatial reversal (SR) task. In terms of VTEs, whereas sham-lesioned animals engaged in more VTE behavior prior to identifying the location of the reward as opposed to repeated trials after it had been located, the lesioned animals failed to show this difference. In contrast, damage to the hippocampus had no effect on acquisition of a VD or on the VTEs seen in this task. For both lesion and sham-lesion animals, adding an additional choice to the VD increased the number of VTEs and decreased the accuracy of choices. Together, these results suggest that the hippocampus may be specifically involved in VTE behavior during spatial decision making.

## Introduction

When a rat makes a decision at a choice point on a maze, it often exhibits side-to-side movements of the head as it looks down each of the alternative routes. This behavior was described in early work by Yerkes (1907), and was subsequently termed vicarious trial-and-error (VTE) by Meunzinger (1938). Meunzinger reported that VTE behavior was associated with learning, and Tolman (1939) later reported that the number of VTEs increased as rats learned to discriminate between a black and a white curtain, and then decreased once this task was well learned. This suggests that VTE behavior may be an index of effortful or deliberative decision-making in the rodent. The purpose of the current experiment was to use this behavior to test how the hippocampus contributes to decision-making behavior.

Recent evidence suggests that the hippocampus may play a central role in VTE behavior. In one experiment, Johnson and Redish (2007) associated VTE behavior with anticipatory firing of hippocampal place cells during a spatial task. They recorded CA3 place cell ensembles in the dorsal hippocampus as rats navigated a multiple T-maze and found that, at the highest cost T-junction, CA3 representations tended to fire in sequence ahead of the rat along the alternative routes, transiently representing future possible routes. These hippocampal sweeps occurred when the rat was displaying VTE behavior, suggesting that VTE behavior may be associated with hippocampal representations of the future. This finding in rodents is consistent with the observation that in humans the hippocampus appears necessary for generating detailed descriptions of imagined future events (Hassabis et al., 2007). Further, recent work has shown that patients with hippocampus damage show fewer “spontaneous revisitations”—looks back to previously viewed stimuli in a computer-based memory task—a behavior that is a potential human analog of VTEs (Voss et al., 2011).

Other evidence of a link between VTEs and the hippocampus is less direct. Hu et al. (2006) found a correlation between neural activity in the hippocampus, as indicated by cytochrome oxidase activity, and VTEs during a visual discrimination (VD) task. However, this correlation was also observed in a control group of animals where rewards and visual stimuli were uncorrelated, suggesting that this relationship may not be specific to learning. It has also been shown that VTE behavior decreases following the systemic administration of an NMDA receptor antagonist (Griesbach et al., 1998; Blumenthal et al., 2011), though this effect may not necessarily be hippocampus-specific.

Conceptually, hippocampal function has been linked to VTE by Amsel (1993), who argued that VTEs are the mechanism for learning spatial information and that this is the function of the hippocampus, as opposed to cognitive mapping. Later, Hu and Amsel (1995) compared VTE behaviors in rats with hippocampus lesions to those of control rats during learning of a black-white VD task. They found that hippocampus lesions impaired learning of the discrimination, and produced a transient deficit in VTE behavior.

This finding, though suggestive, suffers from two limitations. First, the hippocampus lesions in the Hu and Amsel may have had an impact on brain areas beyond the hippocampus, as the lesions were produced electrolytically (Jarrard, 1989, 1993). Second, the task employed—a simple VD task—does not necessarily require the hippocampus (Marston et al., 1993).

To address these difficulties, we produced hippocampus lesions with a more selective neurotoxin, ibotenic acid, and utilized a spatial task which requires the hippocampus and in which CA1 place cells encode intended destination (Ainge et al., 2007; Stevenson, 2011). We reasoned that if the hippocampus is essential for encoding future destinations as well as being involved in generating VTE behavior, then VTEs would be significantly reduced during goal-directed navigation following hippocampus lesions. In contrast, on a non-hippocampus dependent task such as a VD, VTEs would be unaffected. We found that rats with hippocampus damage were impaired in performing a serial spatial reversal (SR) task, and did not show an elevated number of VTEs before finding where the food was located in each session, as seen in control animals. In contrast, hippocampus lesions had no effect on performance or VTEs in a simple VD task.

## Methods

### Subjects and design

Twelve male Lister Hooded rats (Charles River Laboratories, UK), weighing 250–300 g at the start of the experiment, served as subjects for this study. The rats were housed four to a cage in a 12 h light/dark cycle environment and trained during their light cycle. During the experiment, all rats were food restricted to ~ 85% of their free feeding weight and allowed free access to water. All procedures were compliant with the UK [Animals (Scientific Procedures) Act (1986)] and with the European Communities Council Directive of 24 November, 1986 (86/609/EEC) legislation governing the maintenance of laboratory animals and their use in scientific experiments. Rats were first familiarized with the double Y-maze apparatus and task rules, and then underwent a sham surgery or a hippocampus lesion surgery. They were then tested on the spatial task, and finally tested on a VD task.

### Apparatus

**Figure 1:**
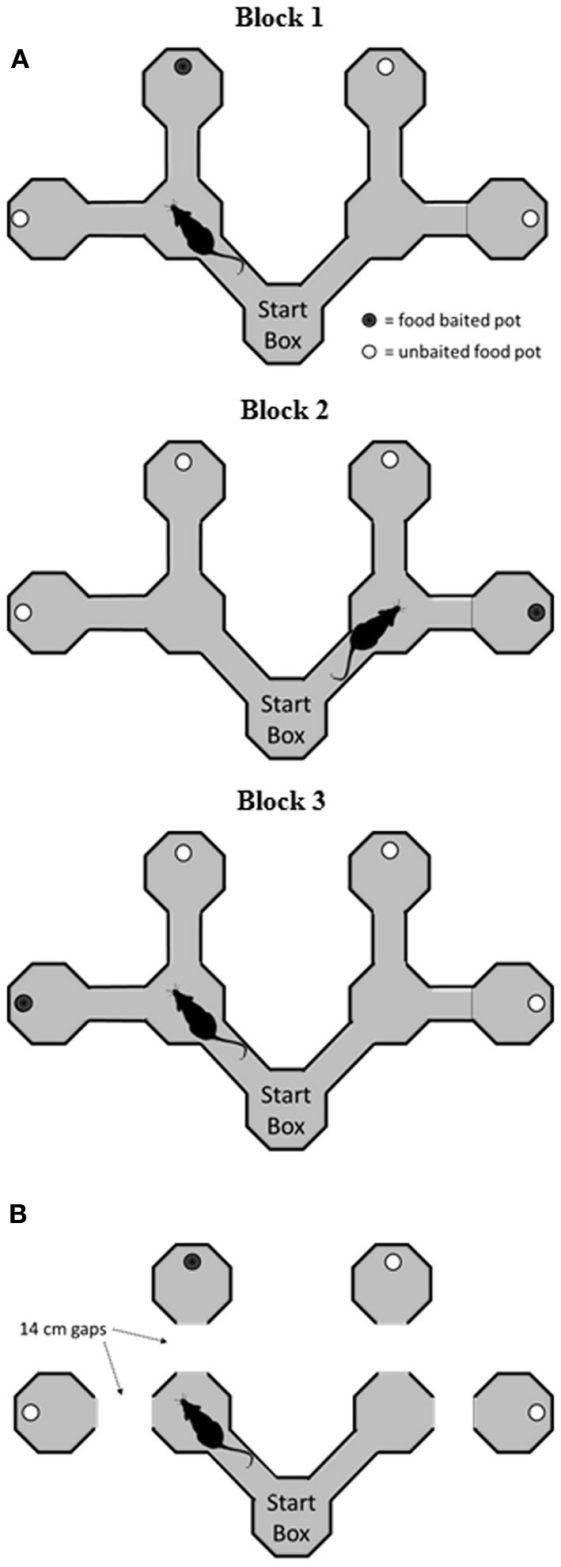
**Schematic of the double Y-maze task. (A)** Before surgery, rats were trained to find food rewards in a goal box at the end of an arm. On each block of trials, the rewards remained in the same goal box, but across blocks they were shifted to different goal boxes. **(B)** Following surgery, rats were tested on the same task, but on a modified version of the maze where the rat had to jump across a small gap to reach the goal box. VTEs were recorded in the start box and at the choice points on each trial.

**Figure 2:**
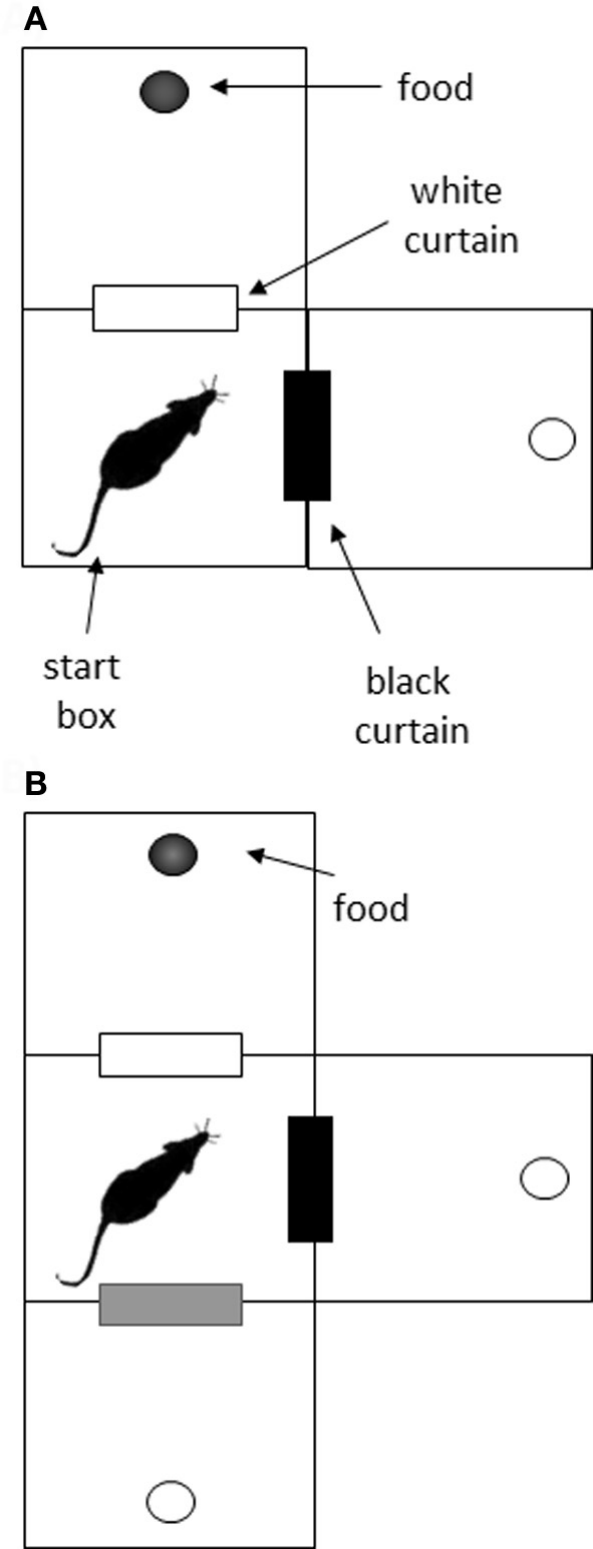
**Schematic of the visual discrimination task. (A)** In the initial two-choice task, rats were placed in the start box, and had to choose between a doorway with a black curtain and one with a white curtain. Food reward was always associated with one color for each rat. **(B)** In the three-choice task, an additional doorway was opened, and it was covered with a gray curtain. The previous association (e.g., white curtain—reward) was maintained in the three-choice situation.

Two apparati were used in the current experiment: a double Y-maze and a VD chamber. The Y-maze was constructed of wood and comprised a start box, two choice boxes, and four goal boxes, connected with alleyways (see Figure 1A). Each of the boxes was octagonal, with 25 cm between opposite edges, and with 30 cm walls. The connecting alleyways were 25 cm long, 8 cm wide, and had 10 cm walls. The maze was painted black, and rested on bar stools within a laboratory room. After surgery, the alleyways before the goal boxes were removed to increase the cost of the decision (Figure 1B). The VD apparatus (Figure 2) initially consisted of three black boxes (49 cm wide, 52 cm high), arranged in an L-shape. One box served as the start box, and in the two-choice condition, it had two doorways, one with a black curtain and one with a white curtain. In the three-choice condition, a third doorway in the start box was opened and covered with a gray curtain. This doorway led to a third choice box.

### Double Y-maze task

The goal of the current study was to assess the effects of hippocampus damage on performance of the task, and not its acquisition. Thus, animals were introduced to the apparatus and the rules of the SR task prior to surgery. Rats were first started on a food deprivation schedule and handled for 5 min/day for 3 days prior to initial maze training. Then rats were trained to run from the start area on the maze and find the location of food rewards in one of the four goal boxes (see Figure 1A). Weetos chocolate cereal loops (Weetabix, Kettering, UK), broken into quarters, were used as rewards. These were placed in a circular bowl in one of the four destination boxes; the remaining three boxes also had food bowls, but the food in these bowls was placed below a metal grate, and was therefore inaccessible. At the start area, there was a choice of two alternative routes, each leading to another junction with two possible routes. These four alternative paths each led to a distinct goal box. Only one goal box was baited on each trial, and the specific goal box that was baited changed between blocks of trials. Rats were placed facing the South wall of the start box at the beginning of each trial to maximize the possibility that any decision was made after the trial had begun. On a trial, if the rat found the food location, it was permitted 10 s to feed before being placed on a nearby platform to await the next trial. If the rat went to an incorrect goal box, a wooden block was placed at the start of the alley leading to the box, and the rat was confined to this area for 10 s before being placed on the platform. The maze arms were wiped down with a detergent soaked cloth after each trial. During training days 1–4, rats were given two blocks of trials per session or 30 min, whichever came first. Thereafter, they were given three blocks per session. At the start of a block, the rats were unaware of the location of the reward. Thus, they were given as many trials as needed to locate the rewarded box. Once the reward location had been sampled, nine additional trials were given with the reward in the same location. The reward location was then changed to another goal box and the next block of trials commenced. Rats were trained until they reached a performance of 80% correct trials over the three blocks (for trials after the baited box had been found) on two consecutive sessions before undergoing surgery.

### Surgery

Animals were anaesthetized with isoflurane (Abbott, UK) and placed in a stereotaxic frame (Kopf Instruments, USA). Anaesthesia was maintained via an inhalation nose cone affixed to the mouth bar on the frame. Under sterile conditions, a midline incision was made and the skull exposed. For the sham animals (*n* = 5), holes were drilled into the skull bilaterally over the hippocampus. The dura was then pierced several times on each hemisphere, a small piece of gelfoam was added to the hole, and the animal's skin was sutured. For the lesion rats (*n* = 7), holes were drilled over the hippocampus and ibotenic acid (Tocris, UK; 10 mg/ml, pH 7.4) was injected at 13 sites in each hemisphere (see Table 1 for coordinates and volumes used). At the end of surgery, rats received subcutaneous injections of small animal Rimadyl (Pfizer Ltd, UK; 0.08 ml/kg bodyweight) as an analgesic. One lesioned animal failed to recover from the anaesthesia, and thus 11 animals were used in subsequent behavioral testing. All rats were given 10 days to recover with unlimited access to food and water, followed by four days of food restriction prior to returning to the experiment.

**Table 1:** **Coordinates for ibotenic acid infusions into the hippocampus**.

**AP (mm)**	**ML (mm)**	**DV (mm)**	**Volume of ibotenic acid (μL)**
2.4	±1.4	3.0	0.05
3.0	±1.4	2.1	0.05
3.0	±1.4	2.9	0.05
3.0	±3.0	2.7	0.10
4.0	±2.6	1.8	0.05
4.0	±2.6	2.8	0.10
4.0	±3.7	2.7	0.05
4.3	±4.0	7.0	0.05
4.9	±3.9	3.5	0.10
4.9	±3.9	7.0	0.10
5.9	±4.3	3.9	0.08
5.9	±5.1	4.5	0.08
5.9	±5.1	5.3	0.08

Hippocampus lesion coordinates.

Following recovery from surgery, rats were given 1–4 sessions on the serial reversal task on the double Y maze as described above until they ran four blocks in a session. Then, testing continued on a modified version of the maze (Figure 1B). On this version, the final alleys leading to the goal boxes were removed so that rats had to make a short jump across a gap to reach a given goal box. The distance of this gap was kept at 14 cm throughout. The rationale for introducing the gap was to increase the cost of making a wrong decision by increasing the effort required to reach the reward, as had been done in early studies on VTE (e.g., Tolman and Ritchie, 1943). Rats were blocked in the goal box for 10 s once again for making a wrong decision. Rats were tested for 16 sessions, where each session consisted of four blocks of trials, each with a different goal box baited. This allowed the rat to experience rewards in all four goal boxes during each session.

The location of the goal box that the rat chose was recorded for each trial, and the percentage of correct choices was calculated as the number of reinforced trials/number of total trials. A VTE was counted at a choice-point if an animal faced one alleyway (A), and then turned its head to face the other alleyway (B). Every obvious A–B or B–A shift was scored as a VTE by the experimenter as it occurred, in agreement with the operationalization of Goss and Wischner (1956). VTEs were counted at both the first and second decision points on the maze. In cases of uncertainty as to whether a VTE had occurred on a given trial, a DVD recording of the trial was consulted.

### Visual discrimination task

Following completion of the double-Y maze task, rats were assessed on a non-spatial VD task. The purpose of this assessment was to determine whether removal of the hippocampus affects VTE behavior in a non-hippocampus-dependent task.

Rats were trained to discriminate between doorways covered with different colored curtains in a two-choice and three-choice environment (Figure 2). In the two-choice task, rats were placed into the center box, facing the corner opposite the curtains. Two doorways led from this box; one was covered with a white curtain and the other with a black curtain. On the other side of each doorway was another box with a food cup. Five of the rats received food (chocolate Weetos) after passing through the doorway with the black curtain, and the other six were reinforced for choosing the white-curtained doorway. The non-reinforced box also contained a food cup, but this was empty. These reinforcement contingencies were maintained throughout training. Rats were given 12 trials a day, and the position of the curtains was switched between three and six times in a pseudo-random manner during these trials to discourage any spatial strategy for solving the task. The only exception to this was on training days 10 and 11, where the curtains were deliberately left in the same place for all trials, to test whether incidental spatial information had any effect on choice accuracy. A choice was recorded if the rat entered or poked its entire head through a curtain. A VTE was recorded if the animal faced one curtain and then faced the alternative curtain within a trial.

Following 20 sessions with the two-choice task, a third choice was added. The motivation here was to see whether increasing the number of options increases the difficulty of the task, and thereby the number of VTEs. The third choice was a doorway covered with a gray curtain, and the chamber this doorway led to did not contain any rewards. The reinforcement contingencies (black curtain S+ or white curtain S+) previously used for the two-choice task remained in place for each rat. Choices and VTEs were counted as above.

Results throughout were analysed using SPSS v.19. For most comparisons, a repeated measures ANOVA was conducted, with group (sham vs. lesion) as a between-subject factor. Where appropriate, linear contrasts are reported.

## Results

**Figure 3:**
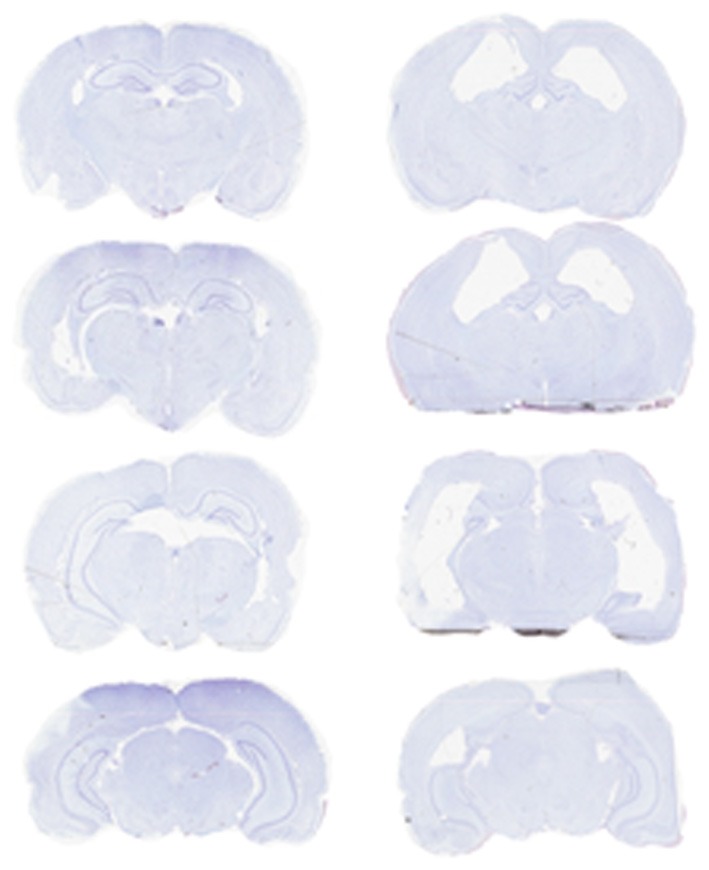
**Photomicrograph of representative sections from a sham-lesioned (left column) and hippocampus-lesioned (right column) animal.** The latter sections are from F3401 (Table 2), and the lesion extent in this subject was close to the average of the group.

Rats required an average of 14 days (range: 6–28) to achieve an 80% correct performance level on the initial double-Y maze task prior to surgery. Six rats then received ibotenic acid lesions of the hippocampus, and five rats received sham lesions. For the lesioned animals, the infusion produced, on average, an 81.7% loss of cells in the dorsal hippocampus and a 37.8% loss of cells in the ventral hippocampus, for an overall loss of 60.2% of cell layers within the structure (see Table 2; Figure 3).

**Table 2:** **Percentage of damage to the cell layers of the dorsal hippocampus, the ventral hippocampus, and the entire structure**.

**Rat**	**Dorsal hippocampus**	**Ventral hippocampus**	**Total hippocampus**
F3409	80.4	62.3	71.5
F3408	92.9	35.9	64.9
F3402	83.5	36.6	60.5
F3401	61.0	57.1	59.1
F3405	87.4	17.1	52.8
F3407	85.0	18.0	52.1
Mean	81.7	37.8	60.2

The lesion extent is expressed as a percentage of the mean size of the cell layer for the sham animals.

### Hippocampus lesions produce a clear and consistent impairment in the spatial reversal task

**Figure 4:**
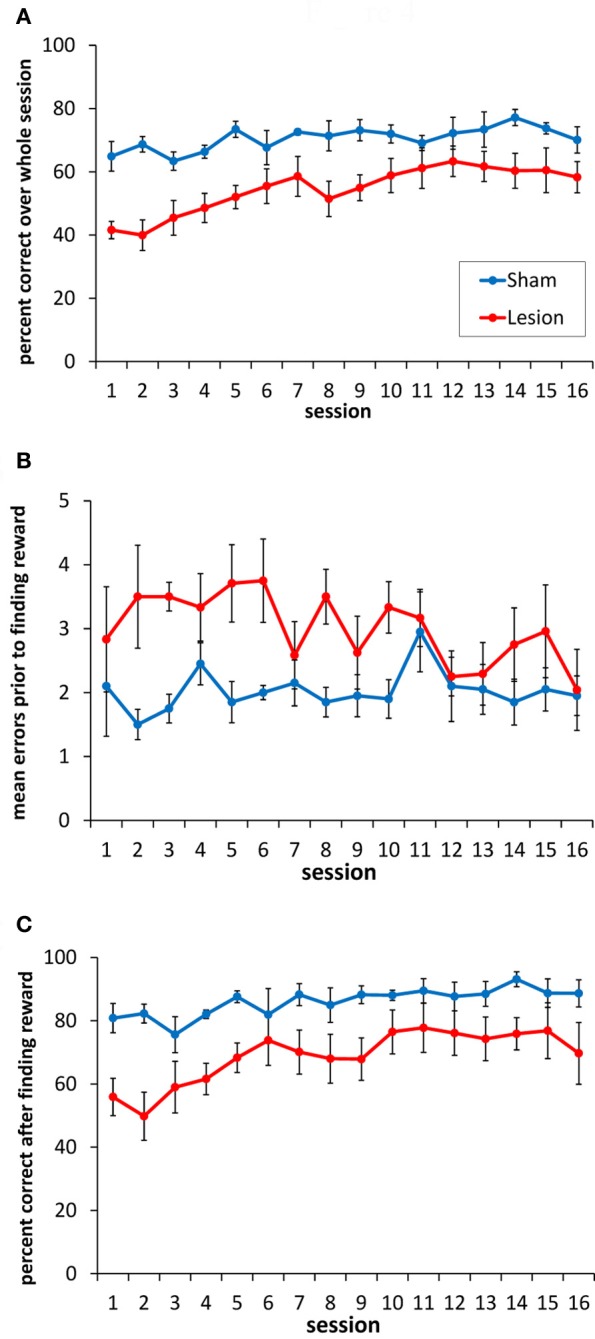
**Performance of the sham-lesioned and hippocampus-lesioned animals on the double Y-maze task. (A)** Animals with hippocampus damage exhibited a consistent impairment in the accuracy of their goal box choices compared to the sham-lesioned animals. **(B)** Animals with hippocampus damage made more errors (non-rewarded box choices) before choosing the rewarded location, compared to the sham-lesioned animals. **(C)** Animals with hippocampus damage made more errors than sham-lesioned animals after the reward location had been found in each block of trials.

Performance of the sham and hippocampus lesion groups on the serial SR (double-Y maze) task is shown in Figure 4. As is evident in Figure 4A, the lesioned animals showed a significant impairment in choice accuracy [*F*_(1, 9)_ = 12.3, *p* < 0.007]. There was also a main effect of testing session [*F*_(15, 135)_ = 4.23, *p* < 0.001], indicating that both groups improved across sessions [linear effect: *F*_(1, 9)_ = 44.8, *p* < 0.001]. The effect of session did not differ between groups [session × group interaction: *F*_(15, 135)_ = 1.24, *p* = 0.247].

To explore the nature of the impairment in more detail, we separated each session into the trials before the reward was found within each block, and the trials after the reward location had been identified within each block. Rats with hippocampus lesions made more incorrect box choices than the sham animals before finding the rewarded location (Figure 4B) [*F*_(1, 9)_ = 7.14, *p* < 0.05], and they continued to make more errors after finding it (Figure 4C) [*F*_(1, 9)_ = 7.9, *p* < 0.05].

### Sham-lesion animals, but not animals with hippocampus lesions, show more VTEs before finding the rewarded locations compared to trials after the reward has been found

**Figure 5:**
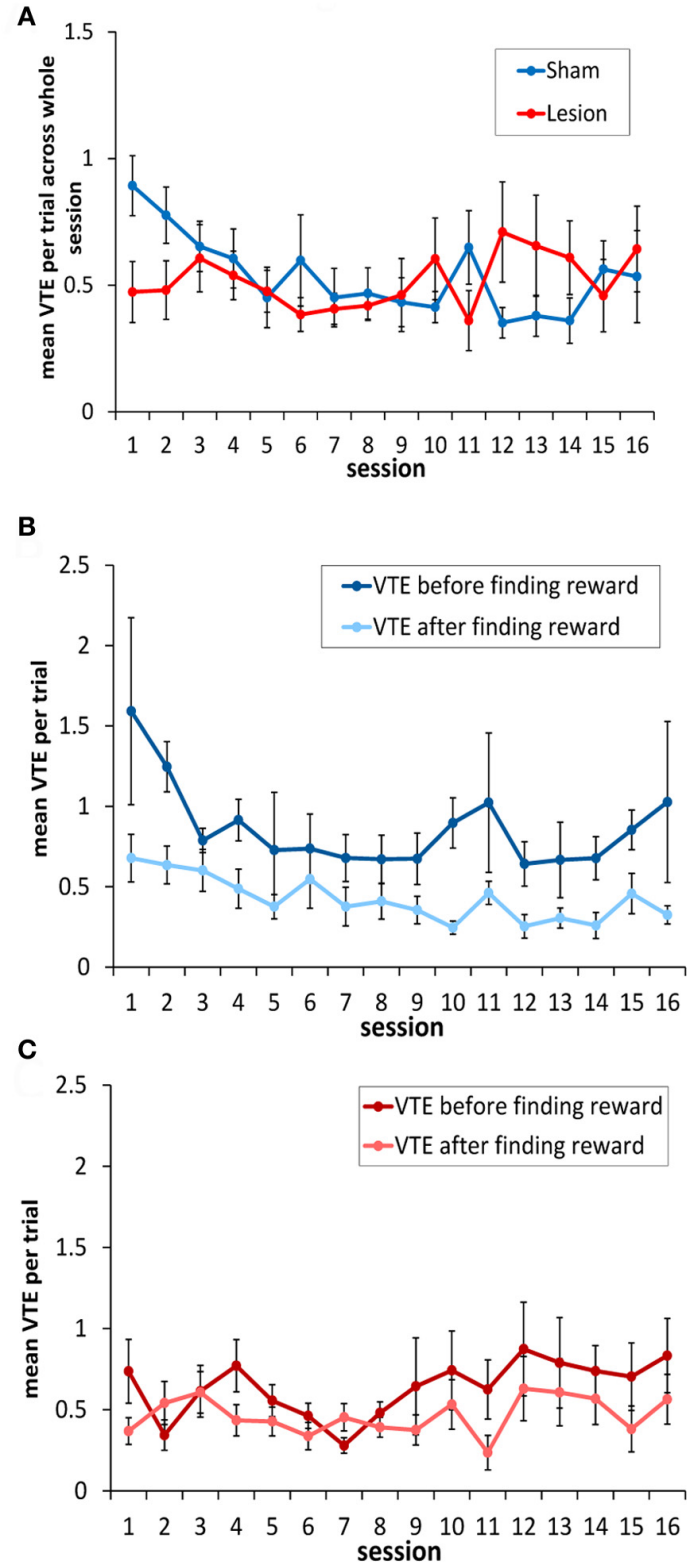
**Vicarious trial-and-errors in hippocampus- and sham-lesioned animal. (A)** With the exception of session 1, animals with hippocampus damage showed a similar number of VTEs to sham lesioned animals. **(B)** Sham-lesioned animals made significantly more VTEs before finding the rewarded goal box in each block, compared to after this location had been identified. **(C)** In contrast, animals with hippocampus damage showed a similar number of VTEs both before the rewarded goal box had been found in each block, and after it had been found.

Across the 16 sessions of testing, there was no overall change in mean number of VTEs/trial [*F*_(15, 135)_ = 1.08, *p* = 0.38], and the sham and hippocampus lesion groups did not differ [*F*_(1, 9)_ = 0.02, *p* = 0.88] (see Figure 5A). The sham and hippocampus lesion groups did differ on an individual session [session × group interaction: *F*_(15, 135)_ = 2.38, *p* < 0.004], and the source of this interaction was a higher number of VTEs/trial in session 1 for the sham-lesioned animals compared to those with hippocampus lesions [equal variances not assumed: *t*_(8.92)_ = 2.49, *p* < 0.04]. In the remaining sessions, the two groups did not differ significantly.

In testing the rats, the experimenters noted that rats tended to show more VTEs before finding the rewarded location as opposed to after it had been found. Once the food had been located, the animals appeared to behave in a less deliberative way, and often made a ballistic return run to the rewarded box. Thus, as in the correct choice data above, the mean number of VTEs/trial was calculated for the trials before the animals encountered the rewarded location in each block and for the trials after it had been encountered. On this analysis, sham animals exhibited significantly more VTEs per trial before finding the reinforced box than after it had been located [*F*_(1, 4)_ = 8.6, *p* < 0.05; see Figure 5B]. In contrast, rats without a hippocampus showed a low level of VTE behavior both prior to finding reward and afterwards, with no significant difference between them [*F*_(1, 5)_ = 3.12, *p* < 0.14; Figure 5C].

**Figure 6:**
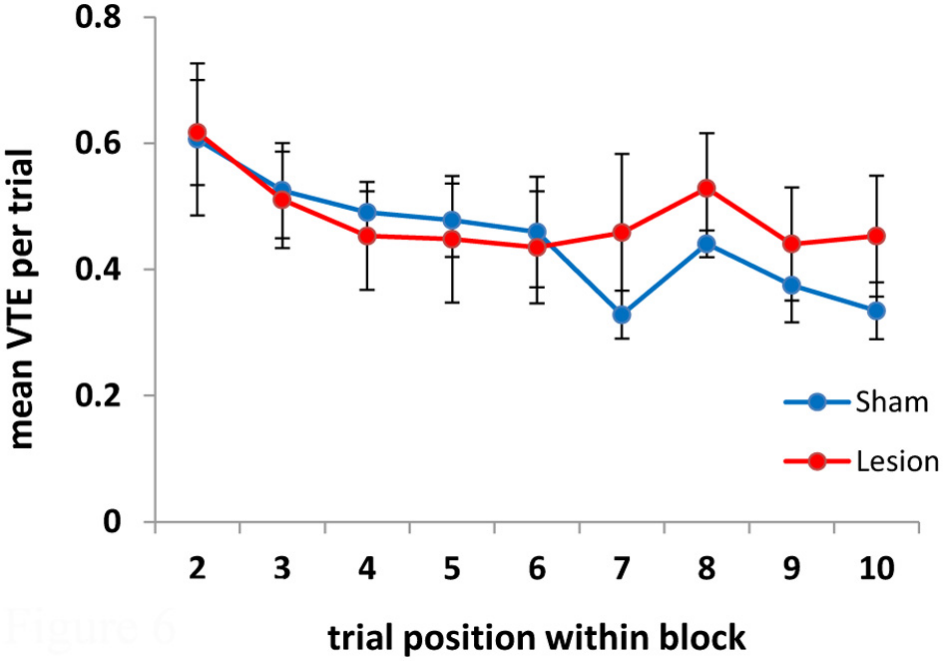
**VTEs as a function of trial within each block.** VTEs decreased over trials within each block. Note that the first trial within each block is not included in this analysis as on the first trial the animals do not know that a given goal box is rewarded until they reach it.

A recent report by Papale et al. (2012) noted that VTEs in a maze-based delay-discounting task occurred with greater frequency in the early trials of each session, which corresponded to trials in which the animals adjusted the delay before receiving reward. As an additional analysis, to see whether this pattern of VTE behavior holds for other tasks, we looked at the mean number of VTEs/trial once the animals had located the reward. The first trial in each block was not considered in this analysis, as VTEs in it occurred before the animal identified the reward location. As is evident in Figure 6, both the sham- and the hippocampus-lesioned groups tended to show more VTEs in the initial trial of each block, as opposed to the subsequent trials [main effect of trial: *F*_(8, 72)_ = 4.86, *p* < 0.001; linear trend: *F*_(1, 9)_ = 10.67, *p* < 0.01]. In this analysis, there was no difference between the two groups [*F*_(1, 9)_ = 0.08, *p* = 0.78] or interaction between groups and trials [*F*_(8, 72)_ = 1.13, *p* = 0.35]. We also found that more VTEs were made on error trials within each block, as opposed to the trial before or after an error [trial type: *F*_(2,18)_ = 5.4, *p* < 0.016; group effect: *F*_(1, 9)_ = 1.62, *p* = 0.24; trial type × group interaction: *F*_(2, 18)_ = 0.98, *p* = 0.39; Pairwise comparisons: error trial vs. preceding trial mean difference = 0.16, *p* < 0.002; error trial vs. next trial mean difference = 0.15, *p* < 0.03].

### Animals with hippocampus lesions are unimpaired in a simple visual discrimination task

**Figure 7:**
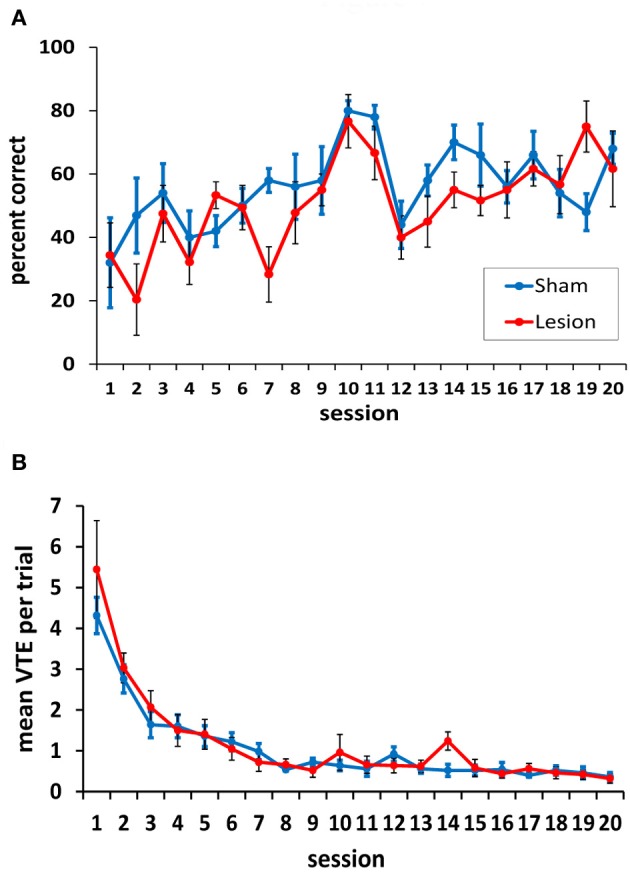
**Performance on the two-choice visual discrimination task. (A)** Both animals with hippocampus damage and sham lesions acquired the visual discrimination, and the two groups did not differ in this acquisition. **(B)** Both the hippocampus- and the sham-lesioned animals showed more VTE behavior in the initial training sessions compared to the subsequent sessions. No differences between the two groups were observed in VTEs.

Following completion of the testing on the spatial task, rats were tested in black/white VD task. Across 20 training sessions, there was no significant difference between the hippocampus- and sham-lesioned animals [*F*_(1, 9)_ = 0.93, *p* = 0.36], and the two groups did not behave differently across sessions [*F*_(19, 171)_ = 1.46, *p* = 0.11]. Both groups improved across sessions [session effect: *F*_(19, 171)_ = 5.78, *p* < 0.001; linear effect: *F*_(1, 9)_ = 54.61, *p* < 0.001; see Figure 7A].

As is evident in Figure 7A, rats performed somewhat better on days 10 and 11, when the curtains were left in the same position across trials. Pairwise comparisons confirmed this; performance on day 10 was significantly better than all other days except day 11 (all *p*-values < 0.05). Day 11 performance was better than 9 other days (1–7, 12, 13; *p*-values < 0.05). This result suggests that the task was somewhat easier when the curtains remained in the same position across trials, and that a spatial strategy may have been helpful in identifying the location of the reward when its position was unchanged. This strategy, however, could not have been used on the other testing days as the position of the curtains was changed 3–6 times in each session.

In terms of VTEs/trial, both groups showed a higher rate of VTE behavior during the initial sessions in the task, but these dropped to a low level by session 8 (Figure 7B). This was confirmed statistically by a significant main effect of session [*F*_(19, 171)_ = 30.74, *p* < 0.001]. The hippocampus- and sham-lesioned groups showed a similar rate of VTE behavior in this task, and this is supported by a non-significant main effect of group [*F*_(1, 9)_ = 0.14, *p* = 0.72] and a lack of an interaction between groups and testing sessions [*F*_(19, 171)_ = 0.83, *p* = 0.67].

It could be argued that the lack of a hippocampus lesion effect on the VD was seen because some of the ventral hippocampus tissue remained. This possibility cannot be fully excluded. However, to assess this qualitatively, we compared the VD performance of the animals with the largest amount of ventral hippocampus damage (59.7% average cell loss) to the animals with the smallest amount (26.9% average). The animals with the largest ventral hippocampus performed at a 67.5% correct level for the last 10 sessions of testing on the VD, while those with smaller damage to the ventral hippocampus performed at a 57.5% correct response level over the same period. This pattern holds even if the full 20 sessions of the VD task are considered (largest ventral hippocampus lesions: 62.8% correct; smallest ventral hippocampal lesions: 44.6% correct).

The average number of VTEs per trial in the initial sessions may appear to be higher in the VD task compared to the SR task. However, we note that all animals were pretrained on the spatial task before surgery. Thus, the post-surgery VTE curves between the two tasks are not necessarily comparable.

### Adding a third choice makes the discrimination more difficult and results in an increase in VTEs

Although no difference between the lesion and sham groups was evident in this VD task, it was of interest to see whether increasing the number of decision options had an effect on performance or VTE behavior. We reasoned that adding a third option (see Figure 2B) would increase the deliberative demands of the task, at least initially.

**Figure 8:**
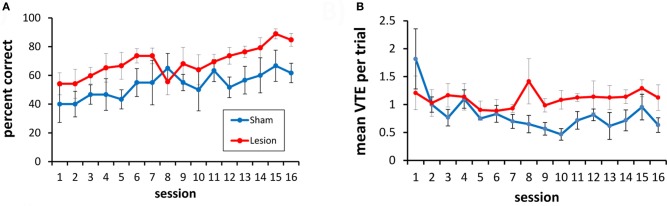
**Three-choice visual discrimination performance and VTEs. (A)** Over 16 sessions of testing, performance on the three-choice discrimination improved, although no significant difference between the hippocampus- and the sham-lesioned animals was observed. **(B)** Mean VTEs per trial over the 16 sessions of testing on the three-choice discrimination.

To test this, rats were given 16 additional testing sessions with a choice between three doorways. In this three-choice discrimination, the lesioned animals appeared to perform at a slightly higher level than the sham-lesioned animals (Figure 8A), although this difference did not reach statistical significance [*F*_(1, 9)_ = 2.49, *p* = 0.15]. Overall, performance improved across training sessions [*F*_(15, 135)_ = 4.94, *p* < 0.001], and this improvement did not differ between the lesion and sham groups [group × session interaction: *F*_(15, 135)_ = 1.1, *p* = 0.36].

For VTEs, there was no difference between the lesion and sham groups [*F*_(1, 9)_ = 3.59, *p* = 0.14; see Figure 8B]. VTEs differed across sessions [*F*_(15, 135)_ = 2.12, *p* < 0.013], but the interaction between group and session did not reach significance [*F*_(15, 135)_ = 1.56. *p* = 0.08].

**Figure 9:**
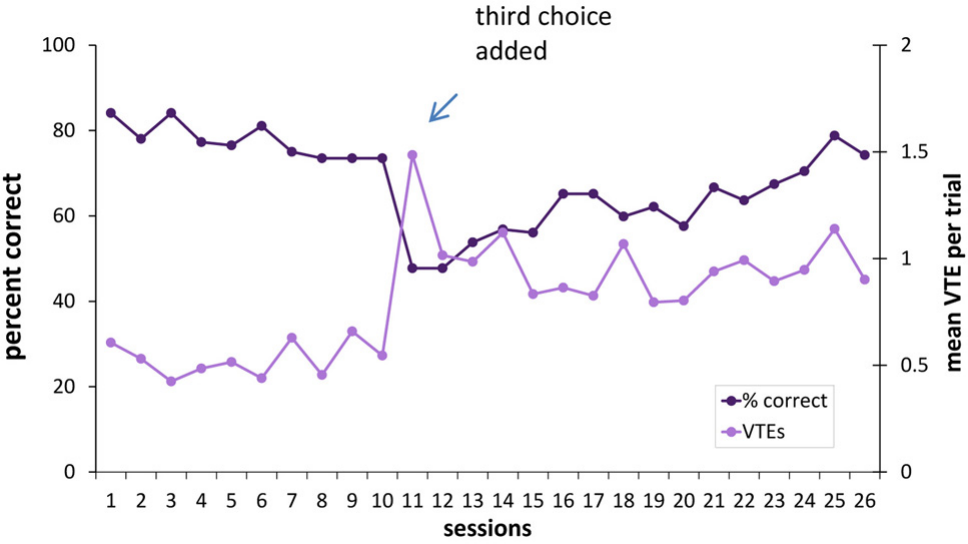
**Percentage of correct responses and VTEs when a third choice is added to the visual discrimination task.** Adding a third choice to the visual discrimination caused a drop in choice accuracy and an increase in VTEs. As the hippocampus- and sham-lesioned animals did not differ in VTEs, they were considered together in this figure.

Although the three-choice task had the same contingencies as the two-choice task (i.e., if an animal was reinforced for choosing the black curtained doorway in the two-choice task it continued to be reinforced for this choice in the three-choice task), the percentage of correct responses was lower in the three-choice environment [*t*_(10)_ = 4.01, *p* < 0.003]. There was a spike in the number of VTEs on the first session with three choices, and an overall higher level of VTEs thereafter [two-choice VTE vs. three-choice VTE: *t*_(10)_ = −9.05, *i* < 0.001] (see Figure 9).

## Discussion

The current experiments examined the contribution of the hippocampus to VTE behavior. Animals with damage to the hippocampus exhibited a consistent impairment in correct responding on a spatial memory task, and exhibited a similar level of VTEs both before identifying the reward location and after finding it. In contrast, sham-lesioned rats showed a higher level of VTE behavior on trials before finding the reward location, as opposed to the trials after it had been found. On a VD task, hippocampus lesions had no effect on choice accuracy or on VTE behavior.

The observed results, with more neuronal-selective lesions than used in the previous study by Hu and Amsel (1995), provide partial support for their findings. On the one hand, we failed to find any effects of hippocampus damage on VD learning or VTE behavior, whereas Hu and Amsel reported an impairment in the number of rewards obtained, and a transient impairment in VTE behavior. However, others have shown that the hippocampus is not necessary for simple VD learning when lesioned with ibotenic acid (e.g., Marston et al., 1993). Thus, it is possible that the effects seen by Hu and Amsel were due to differences in the techniques used for removing the hippocampus.

On the other hand, in a spatial task on which rats with hippocampus damage were impaired, we found a small but potentially important difference between lesioned and sham-lesioned animals in VTEs which may contribute to the significant impairment in choice accuracy in the former. Rats with an intact hippocampus appear to exhibit VTEs as they explore the environment and attempt to identify which of the four goal locations are baited in a given session. This is in line with results from Papale et al. (2012), who found that the best explanation for VTEs was related to information gathering via searching alternative choices. Once the baited location is found, the need for exploration diminishes, as do the VTEs. Rats with hippocampus damage, in contrast, appear to show fewer exploratory VTEs before finding the rewarded location, and simply run down the first alleyway they encounter.

In interpreting the differential effects of the hippocampus lesions on the SR task and the VD task, however, we note several caveats. First, there were structural differences in the apparati for the SR and VD tasks that may have influenced VTE behavior. Specifically, the SR task was on an elevated maze with gaps before the final decision points, whereas the VD task was run on a floor-based apparatus, without gaps at the decision points. Second, the SR task was trained before surgery, whereas the VD task was only experienced after surgery. Thus, we cannot preclude the possibility that the hippocampus is involved in VD learning, but that the task can be learned utilizing other brain structures when the hippocampus is damaged. Likewise, we cannot rule out the possibility that the differential sensitivity of the two tasks reflects pretraining, and not task differences. The current results, however, are consistent with previous studies showing that hippocampus lesions do not impair simple VD learning (Kimble, 1963; Marston et al., 1993; Murray and Ridley, 1999). Also, we have previously found that hippocampus lesions impair acquisition of a comparable SR task trained only after surgery (Stevenson, 2011). Thus, the pattern of results that we have obtained is consistent with spatial tasks being more sensitive to hippocampus damage than simple VD tasks, though we acknowledge that demonstrations of impairments in the latter do exist (e.g., Hu and Amsel, 1995).

A final interpretational caveat for the current findings is that the VD task was trained after completion of the SR task. Thus, the lack of effect in the former might reflect the different amount of time since the lesion, or an interaction between being performing one task and then acquiring a second task. Again, the possibility cannot be precluded. Taken together, although we argue that the differential effects of hippocampus damage observed in the current study reflect differing task demands, we note that alternative interpretations are also possible.

Several additional observations on VTEs are of note from these experiments. First, we have found that in the VD task, animals with hippocampus damage showed the same number of VTEs as sham-lesioned animals. This indicates that VTEs can be driven by brain structures other than the hippocampus. Second, in the acquisition of the VD task, VTEs started at a relatively high-level, and then diminished across sessions. In parallel, choice accuracy increased. The pattern of results is consistent with the notion that VTE behavior aids learning, as argued by Tolman (1939). However, the observed results are markedly different from the parallel increase in choice accuracy and VTEs observed by Hu et al. (2006) on a Y-maze VD task. In their study, VTEs decreased for the first eight sessions of training, much as we have observed. Thereafter, however, VTEs increased. The reason why VTEs in our task remained low after the initial sessions of training while in the Hu et al. task they increased is unclear. Third, surprisingly, simply adding a choice to a well-learned discrimination causes an increase in VTEs, and a decrease in performance. Thus, the animals appear to treat this as a new learning situation. It is possible that the observed increase in VTEs aids in some way the acquisition of “new” discriminations. If so, one may speculate that the higher level of VTEs in the three-choice situation would decrease had we continued testing. Finally, the trend toward lower performance in the sham-lesioned animals in the three-choice discrimination may reflect their attempts to use a spatial strategy to identify the location of the reward. Animals with hippocampus lesions, in contrast, may have benefited from an inability to use spatial information, and thus more readily used the correct stimuli—the visual discriminada—to identify the location of the reward.

The current results provide some support for the findings that the dorsal hippocampus contributes to VTE behavior and the representation of future options (e.g., Johnson and Redish, 2007). However, it should be noted that the performance of the rats with hippocampus damage in the current study, while significantly lower than sham-lesioned animals, was nonetheless consistently above chance levels. The subtlety of the lesion effects on VTE performance may therefore reflect the use of a task that can be solved in part by using circuitry outside the hippocampus. Alternatively, animals may show VTEs for different reasons, only some of which relate to spatial deliberations (Dudchenko et al., 2013; Papale et al., 2012).

In the current study, the infusions of ibotenic acid produced more damage in the dorsal hippocampus than in the ventral hippocampus. Thus, we cannot preclude the possibility that larger ventral lesions would have yielded greater effects on VTEs and spatial behavior. However, it should be noted that the VTE/hippocampus sweep association reported by Johnson and Redish (2007) was identified in the dorsal hippocampus. Also, in the current study, as has been reported previously (Moser et al., 1993, 1995; Hock and Bunsey, 1998; Bast et al., 2009), damage to the dorsal hippocampus was sufficient to produce a clear and consistent impairment in flexible spatial memory. Lesions of the ventral hippocampus, in contrast, do not impair spatial memory under many conditions (Moser et al., 1995; Bannerman et al., 2003; Pothuizen et al., 2004). It remains possible, however, that the remaining ventral tissue in the hippocampus was sufficient to maintain intact VD performance, whereas the impairment on the SR task was a consequence of damage to the dorsal hippocampus. Two points argues against this. First, previous work has suggested that the hippocampus is not essential for simple VDs. For example, Marston et al. (1993) found that ibotenic acid lesions of the hippocampus—which appeared to include the ventral hippocampus—had no effect on the acquisition a conditional VD task. More recently, Knutson et al. (2012) found that humans with hippocampus damage were unimpaired on easier VDs, and that impairments on more complicated discriminations were attributed to memory capacity limits, as opposed to impaired discriminative abilities *per se*. Second, in the current study, animals with larger ventral lesions actually performed at a somewhat higher level than those with less damage to the ventral hippocampus. Thus, it appears more likely that the hippocampus is not essential for the type of simple VD employed in the current experiment.

## Summary

The current study makes three contributions. First, it shows that selective lesions of the hippocampus, damaging on average 81.7% of the dorsal hippocampus and 37.8% of the ventral hippocampus, do not abolish the capacity to make VTE responses, even in a task where these lesions are sufficient to produce a performance impairment. However, VTEs are reduced in animals with hippocampus lesions prior to their identification of the reward location, suggesting that this brain structure may contribute to VTEs associated with deciphering the reward contingencies in the environment. Second, we show that hippocampus damage has no effect on choice accuracy or VTEs in a VD task trained following surgery. Finally, we found that adding a third choice to a well-learned two-choice VD resulted in a decrease in choice accuracy and a concomitant increase in VTEs. This suggests that VTEs are a potential marker of new learning situations.

### Conflict of interest statement

The authors declare that the research was conducted in the absence of any commercial or financial relationships that could be construed as a potential conflict of interest.
